# Supplementary material for: Inferring processes underlying B-cell repertoire diversity
Source: Philos Trans R Soc Lond B Biol Sci. 2015 Sep 5;370(1676):20140243. doi: 10.1098/rstb.2014.0243 (PMC4528420; doi:10.1098/rstb.2014.0243)
Supplement: Supplementary material [file rstb20140243supp1.pdf]

Inferring processes underlying B-cell repertoire diversity – SI

Yuval Elhanati*,[1] Zachary Sethna*,[2] Quentin Marcou,[1] Curtis G. Callan Jr.,[2] Thierry Mora,[3] and Aleksandra M. Walczak[1]

[1] *Laboratoire de physique théorique, UMR8549, CNRS and École normale supérieure, 24, rue Lhomond, 75005 Paris, France**

[2] *Joseph Henry Laboratories, Princeton University, Princeton, New Jersey 08544 USA*

[3] *Laboratoire de physique statistique, UMR8550, CNRS and École normale supérieure, 24, rue Lhomond, 75005 Paris, France*

Inference of alleles and their chromosome distribution

Our method relies on matching read sequences to substrings of genomic V, D and J gene sequences. There is, however, some degree of allelic diversity in these genes, and we initially have no idea which alleles are carried by the individual whose sequences are being analyzed. Some information about common alleles for these genes in the human population can be found in the IMGT database [1], but there is no guarantee that the alleles we are actually faced with are in the database. If we do not have in hand the correct genomic sequence for our individual subject, we will incorrectly identify mismatches between the read and the genomic sequence as being due to sequence error or, in the right context, hypermutation. We do not expect the numerical impact on our analysis of this issue to be large, but since it is an identifiable source of error, it would be desirable to eliminate it, if at all possible, by somehow acquiring accurate genomic sequence for the alleles that are actually present, individual subject by individual subject. We have developed an automated multi-step allele inference process that does precisely this.

As a first step, using naive nonproductive sequences, we run an initial alignment using genomic data derived from a reference genome (in other words, using a unique sequence for each V, D and J gene). After the initial alignment has been completed, we assign to each read in the data set the V, D and J gene with the highest alignment score (this score tries to minimize mismatches and maximize the length of the read that is identified as of genomic origin). Note that we always carry out this procedure on reads in the naive data set so as to avoid the confusing effect of somatic hypermutation. For each gene, we collect all the read subsequences that were assigned to that gene. Working on the collection of strings associated with a particular gene, we then carry out a procedure that sorts these strings into subgroups such that, within each subgroup, every member is a proper substring of the same maximal length string. Each of these maximal length strings typically can be aligned to the reference gene sequence in such a way that it differs from the reference sequence in only a few positions. We typically find that one or two of these subgroups have many more members than any of the others, and we use the maximal string associated with them to construct the individual subject's allele(s) for the gene under consideration. Basically, we extend the maximal string to the full extent of the gene using the reference genome sequence. This defines two alleles per individual for each gene and yields an interim genomic dataset. This procedure obviously cannot detect allelic variation outside the read "window", so these are not true biological alleles, but they capture all the allelic variation that is relevant to our inference procedure. This procedure defines a set of allelic variants of the V, D and J genes appropriate to the individual subject being analyzed. Of course the procedure has to be done independently for each new subject, but the computational overhead of this step is not too heavy.

At this stage it is clear if a particular allele is homozygous or heterozygous, but we have no idea how the heterozygous alleles are associated on two chromosomes. To explore this issue, we use the interim genomic dataset derived through the procedure described above to infer the generative probability distribution responsible for the sequence data set we are working with. One of the outcomes of our probabilistic approach is an evaluation of the joint probability distribution $P(Vallele, Dallele, Jallele)$ for producing any triplet of VDJ alleles. From the organization of the different alleles on two different chromosomes, some V-D, D-J and V-J allele associations are impossible because the recombination machinery works on one chromosome or the other at a time, never both. Given our probabilistic approach, this should be reflected in a lower probability for inappropriate V-D-J triplets in the joint $P(Vallele, Dallele, Jallele)$ probability of the model. We exploit this fact to reconstruct the chromosomal organization and remove wrongly inferred alleles as follows: each gene with two alleles is assigned a two state variable: each gene is marked as either heterozygous or homozygous. At this point, based on the initial assignment described above, each gene that has two candidate alleles is marked as heterozygous. An iterative procedure described below re-assigns the homo/heterozygosity parameters. If the gene is marked as heterozygous, the allele is assigned to one of the two chromosomes, with the constraint that two

---

*These two authors contributed equally

alleles of the same gene cannot lie on the same chromosome. If the gene is marked as homozygous, one of the alleles is "real", while the other is erroneous (again with the constraint that the two alleles of a given gene must be in two different states - real or erroneous.). Finding the chromosomal organization entails doing a search to find the values of these parameters that minimize the net probability (derived from the $P(Vallele, Dallele, Jallele)$ distribution) of recombination scenarios involving V, D or J alleles that do not lie on the same chromosome.

In practice, all genes with two alleles are initially taken to be heterozygous and all alleles are assigned randomly to a chromosome. After initialization, a gene is chosen at random and the probability of scenarios violating the chromosomal organization is computed for the four possible states of the two alleles of this gene (heterozygous - chromosome 1, heterozygous - chromosome 2, homozygous - real allele, homozygous - erroneous allele) given by the previously defined two state variables. A change in the assignment of these parameters is accepted only if it decreases the probability of erroneous recombination events. This step is iteratively repeated until no further change is possible. This procedure is ensured to converge to a local minimum. Repetitions of this procedure starting from randomly chosen initial states always converge to the same final state, and we conclude that only one global minimum exists.

For the purposes of this paper, we use the results of the procedure just described to identify spurious alleles , leaving a final, cleaned-up, list of alleles for the given individual subject. Summary information about the genes that this procedure declares to be heterozygous (for individual subject A) is presented in Tables S1 and S2 below. We have used this allelically improved, individual specific, genomic data set to carry out the model inference procedure described in the main text. For reasons of computational convenience, however, the model that we inferred is in fact rather simpler than the one used (as described above) to infer the genomic alleles. To be specific, instead of using the gene choice factor $P(Vallele, Dallele, Jallele)$, we used the less complex structure $P(V)P(D,J)P(Vallele|V)P(Dallele|D)P(Jallele|J)$ (distinguishing between gene and allele of same gene). For most of the quantities we compute, the less complex model gives almost the same results as the fully allelic model. However, only the latter model will be able to capture the correlations that arise from chromosomal organization of alleles and, while the associated effects are not large, they are worth tracking. Evidence that the fully allelic model for gene choice really captures all the correlations present in the data is presented in Fig. S3. That figures displays correlations (as quantified by mutual information) between various model variables and compares the values derived from sequence data with the values derived directly from the model (the fully allelic one) inferred from the same data. The match between the two is nearly perfect (although the data does show evidence of a few very weak correlations that the model structure cannot incorporate), which we take as a validation of our hypothesized model structure. Simpler model structures in general show a significantly less impressive match.

[1] Giudicelli V, Chaume D, Lefranc MP (2005) IMGT/GENE-DB: a comprehensive database for human and mouse immunoglobulin and T cell receptor genes. *Nucleic Acids Research* 33:D256–D261.

TABLE I: Heterozygous V allele information (Individual A)

Gene	Allele #	Overall Usage	Fractional Allele Usage	Mismatch Positions from start (from end)
IGHV1-3	1	0.012	0.481	187 (-109), 267 (-29), 272 (-24)
-	2	0.012	0.519	-
IGHV1-69	1	0.011	0.27	214 (-82), 220 (-76)
-	2	0.029	0.73	-
IGHV1-8	1	0.0099	0.533	210 (-86)
-	2	0.0087	0.467	-
IGHV2-26	1	0.0044	0.634	258 (-43), 295 (-6)
-	2	0.0025	0.366	-
IGHV3-20	1	0.0074	0.51	283 (-13)
-	2	0.0071	0.49	-
IGHV3-23	1	0.033	0.627	180 (-116), 219 (-77)
-	2	0.019	0.373	-
IGHV3-33	1	0.0012	0.85	276 (-20)
-	2	0.0002	0.15	-
IGHV3-47	1	0.0021	0.645	243 (-48)
-	2	0.0012	0.355	-
IGHV3-53	1	0.011	0.582	214 (-79), 261 (-32)
-	2	0.0081	0.418	-
IGHV3-69-1	1	0.0018	0.337	170 (-123), 213 (-80)
-	2	0.0036	0.663	-
IGHV3-71	1	0.0071	0.66	278 (-24), 282 (-20)
-	2	0.0037	0.34	-
IGHV3-9	1	0.045	0.538	200 (-98), 272 (-26)
-	2	0.039	0.462	-
IGHV4-38-2	1	0.012	0.857	267 (-27)
-	2	0.002	0.143	-
IGHV4-59	1	0.01	0.469	261 (-32)
-	2	0.011	0.531	-
Total Heterozygous V Usage		0.32		

TABLE II: Heterozygous D and J allele information (Individual A)

Gene	Allele #	Overall Usage	Fractional Allele Usage	Mismatch Positions from start (from end)
IGHD1-14	1	0.00051	0.339	12 (-5)
-	2	0.001	0.661	-
IGHD1-7	1	0.0043	0.802	4 (-13), 12 (-5)
-	2	0.0011	0.198	-
IGHD2-2	1	0.05	0.244	29 (-2)
-	2	0.15	0.756	-
IGHD2-21	1	0.017	0.455	19 (-9)
-	2	0.02	0.545	-
IGHD4-4	1	0.0021	0.81	11 (-5)
-	2	0.00048	0.19	-
IGHD5-5	1	0.0053	0.721	17 (-3)
-	2	0.0021	0.279	-
Total Heterozygous D Usage		0.26		
IGHJ6	1	0.0087	0.297	18 (-45), 19 (-44), 20 (-43), 36 (-27)
-	2	0.021	0.703	-
Total Heterozygous J Usage		0.029		

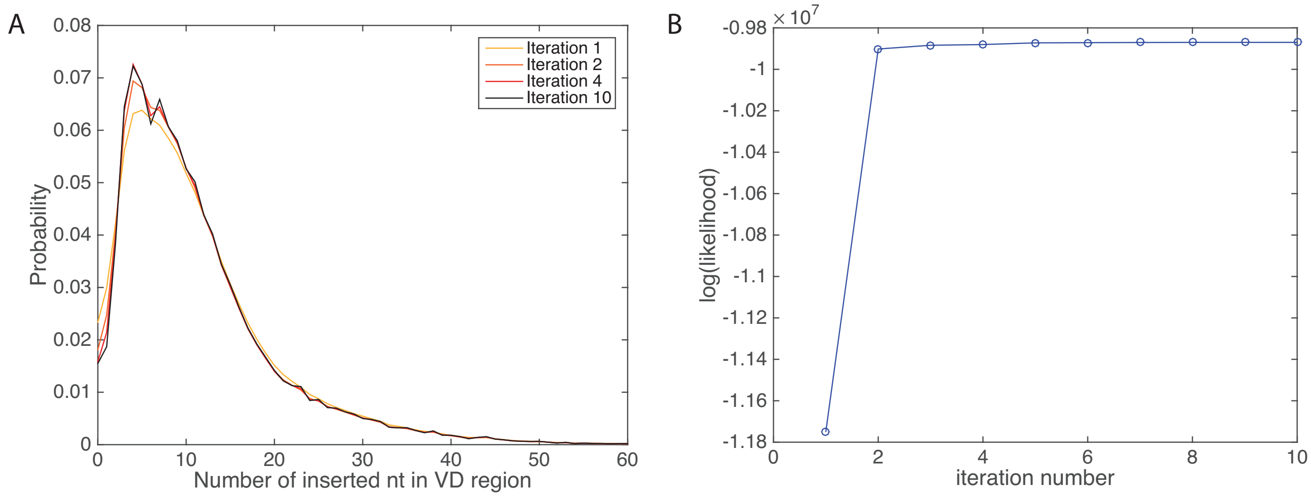

FIG. S1: Convergence with successive iterations of the expectation maximization procedure for inferring the pre-selection model P_{pre}. A: The VD insertion distribution for individual A over ten iterations is displayed. Convergence to a stable distribution is complete after only a few iterations. B: Log-likelihood of the data under the model, as a function of iteration number. The algorithm converges rapidly to its maximum likelihood solution.

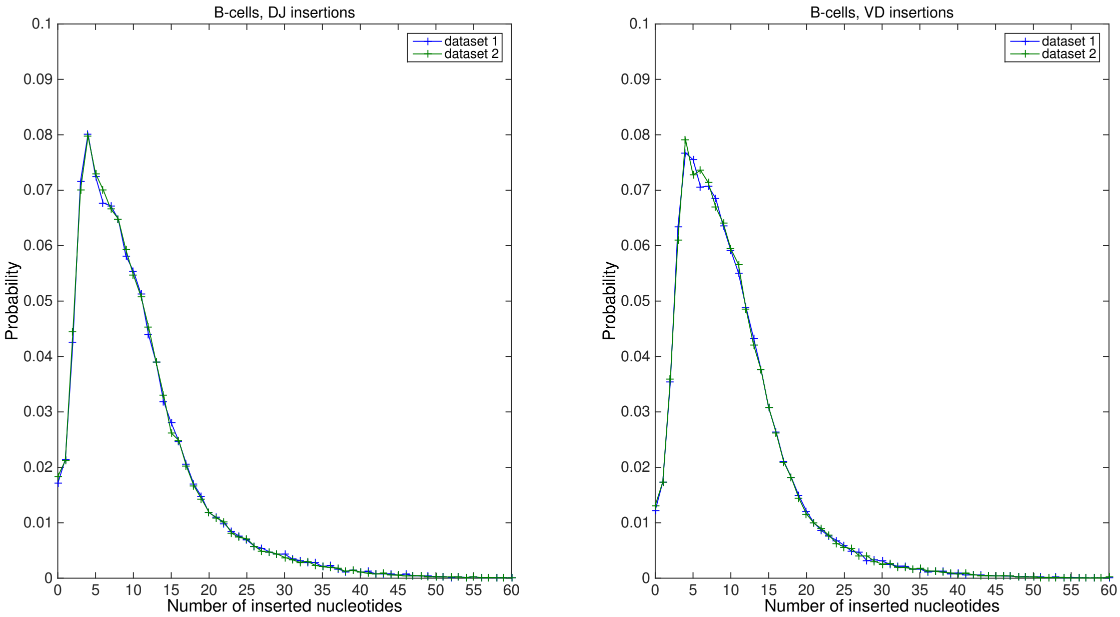

FIG. S2: Robustness of model inference to statistical noise in the pre-selection model P_{pre}. Model distributions inferred from disjoint data sets of 200K sequences from individual A are compared. The distributions are essentially indistinguishable.

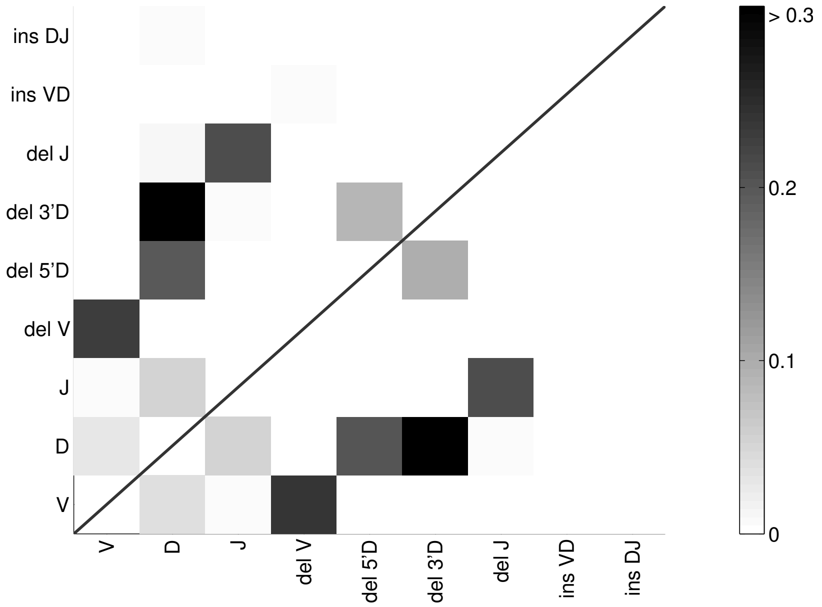

FIG. S3: Matrix of data-derived mutual information between variables describing the VDJ recombination scenario. The pairwise mutual informations derived from the data (resp. directly from the generative model) are displayed above (resp. below) the 45 degree straight line The assumed structure of the recombination model implies that there is no correlation between certain variable pairs, such as VD and DJ insertions. The observed correlations in the sequence data for such variable pairs are zero within statistical noise, indicating that the model structure is in good accord with the correlations present in the data.

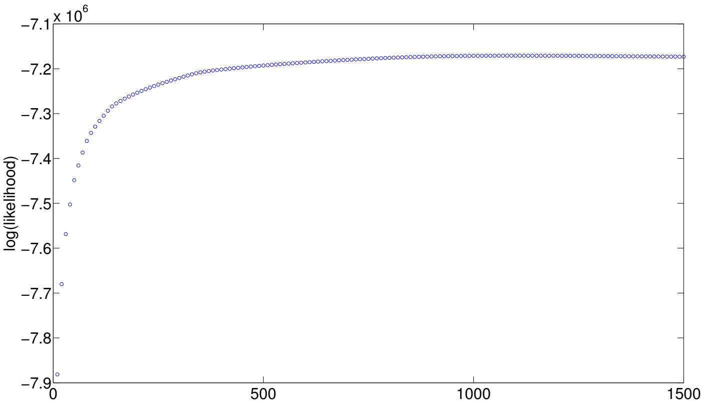

FIG. S4: Convergence of the expectation maximization procedure for the selection model Q. Plotted is the log-likelihood of the data under the model - $\sum_{\sigma} \log[\sum_{\mathrm{V,J}} \mathrm{Q}(\sigma, \mathrm{V}, \mathrm{J}) \mathrm{P}_{\mathrm{pre}}(\sigma, \mathrm{V}, \mathrm{J})]$, as a function of iteration number. The algorithm converges to a stable maximum likelihood solution.

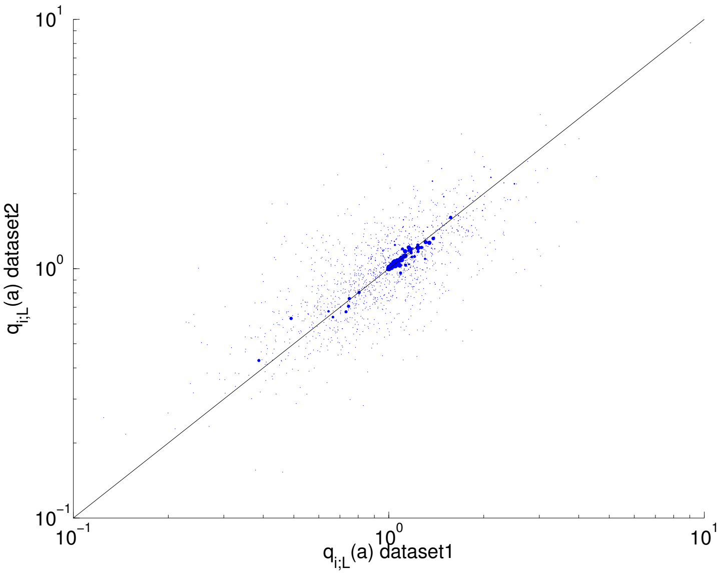

FIG. S5: Robustness of the model inference to statistical noise for the selection model Q. Each point in the scatter plot corresponds to one position/length/amino-acid combination. The size of each point corresponds to product of usage probability of this combination in the two datasets. Combinations with negligible usage are not shown. The points cluster around the line of unit slope, with the clustering being very tight for the points with high usage probability. The scatter around the identity line increases with decreasing usage probability, as is expected for sample noise.

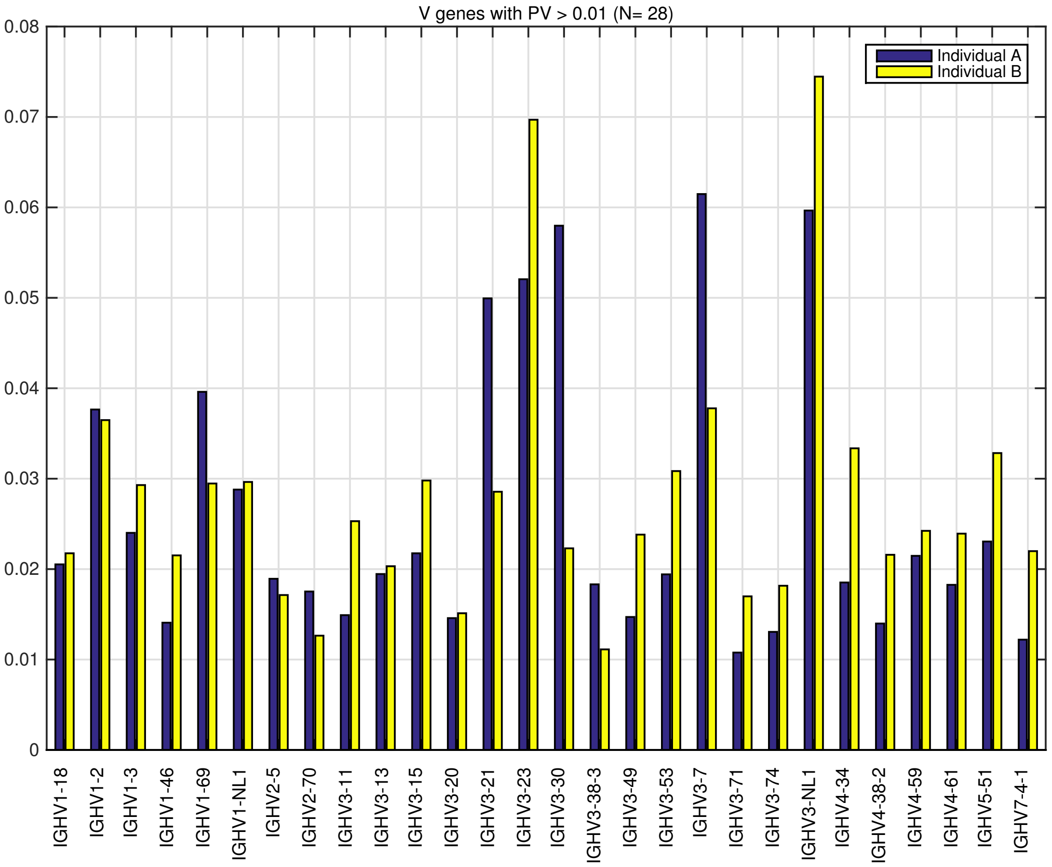

FIG. S6: V gene usage frequencies in VDJ recombination as inferred from naive nonproductive sequence repertoires for individuals A and B. Usage probability varies widely between genes and this plot displays only those V genes with usage frequency greater than .01 for both individuals. Less than half of all V genes pass this filter. The variation between individuals, even for genes with the highest usage rates, is quite substantial.

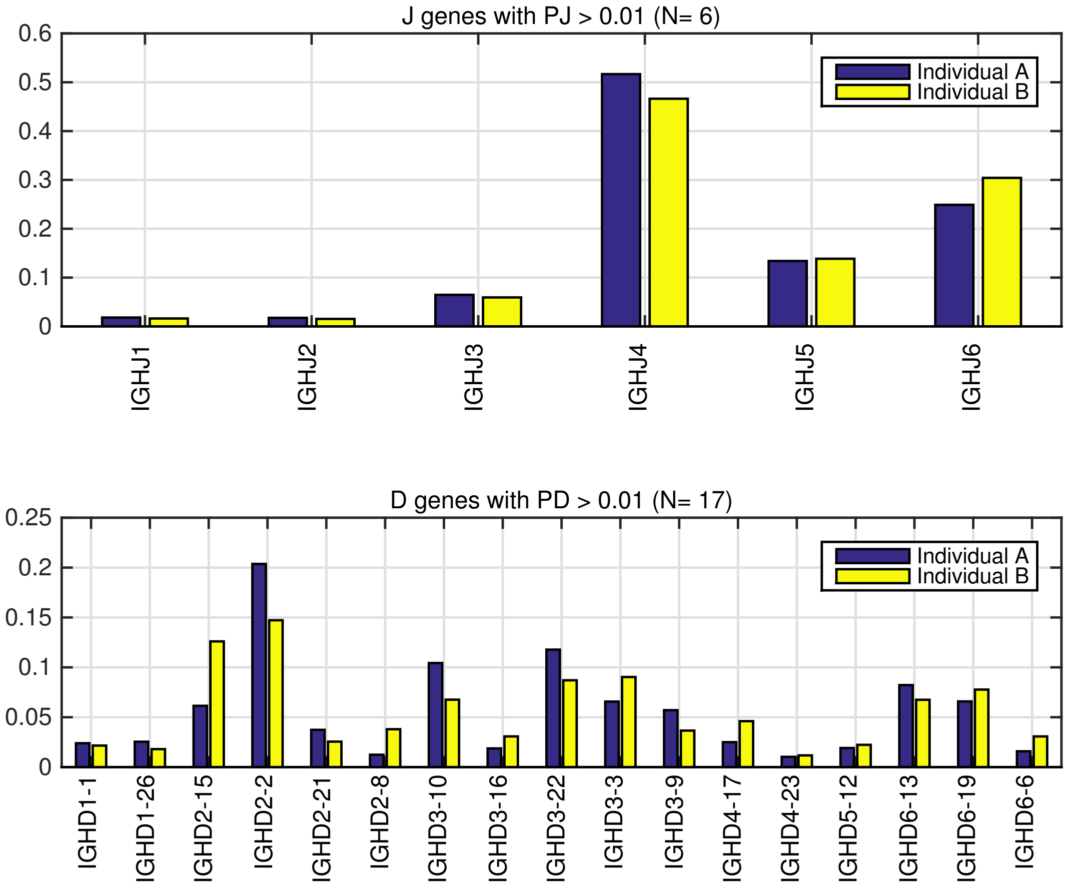

FIG. S7: D and J gene usage frequencies in VDJ recombination as inferred from naive nonproductive sequence repertoires for individuals A and B. Once again the plot only displays genes with usage frequency greater than .01 for both individuals. Less that half of the D genes (but all of the J genes) pass this filter. The variation between individuals of D gene usage (but not of J gene usage) is quite substantial.

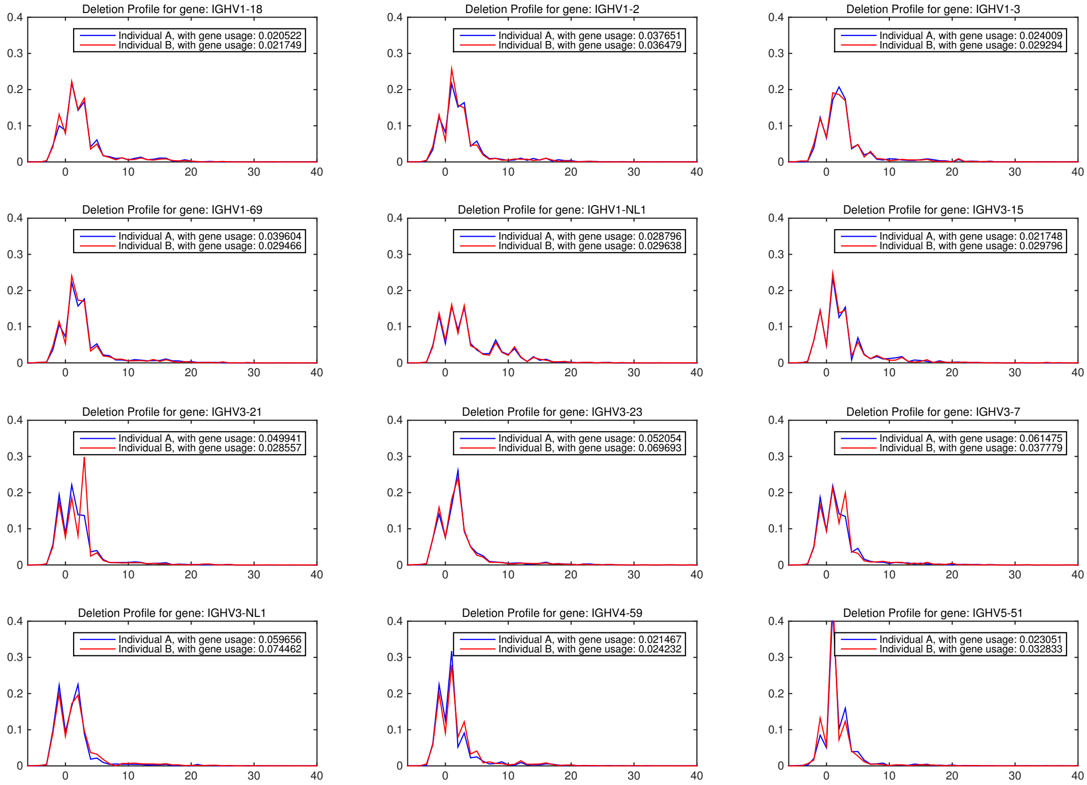

FIG. S8: Gene specific deletion profiles for a few frequently used V genes for individuals A and B (the profile for each of the two individuals is displayed separately on the same plot). Negative deletions account for the occurrence of "palindromes" of different length: when there is no deletion, some length of double-stranded DNA at the beginning of the gene can be "unfolded" into a single-stranded palindrome that then gets filled in as double-stranded DNA before the insertion machinery acts. The deletion apparatus is very sensitive to sequence context, and the deletion profile is quite variable from gene to gene. Remarkably, the deletion profile is nearly identical between the two subjects.

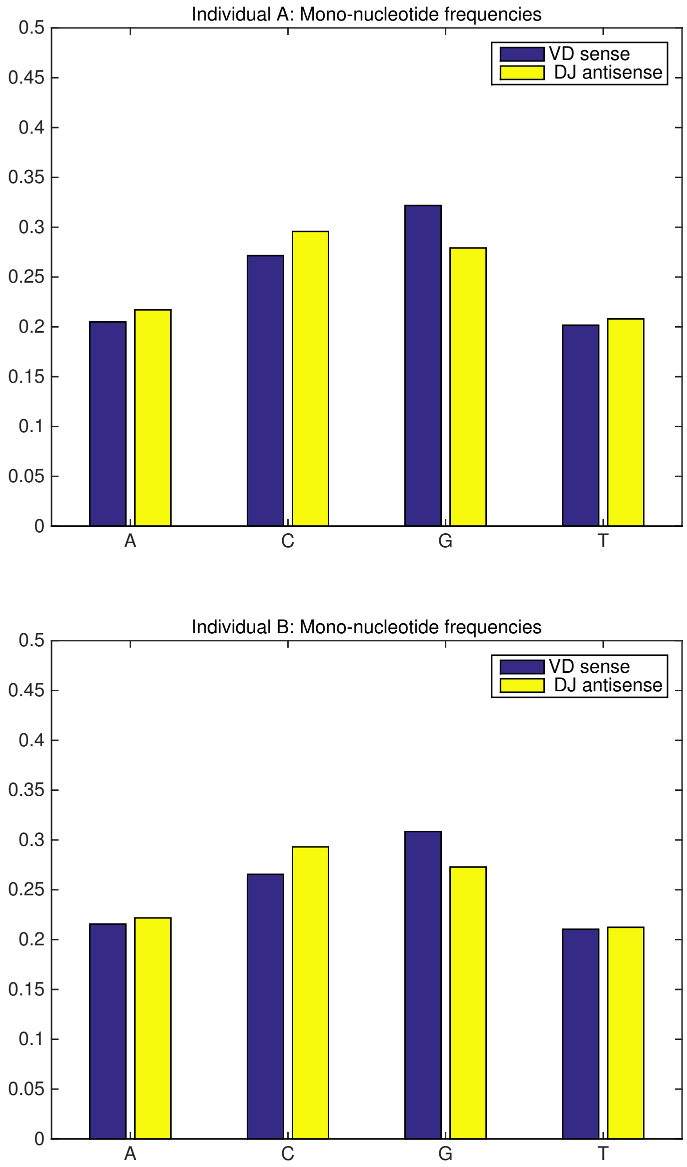

FIG. S9: Mononucleotide utilization frequency for insertions. Top panel - individual A, Bottom panel - individual B. A simple mononucleotide Markov model for generating VD and VJ insertions does not describe their sequence statistics with complete accuracy.

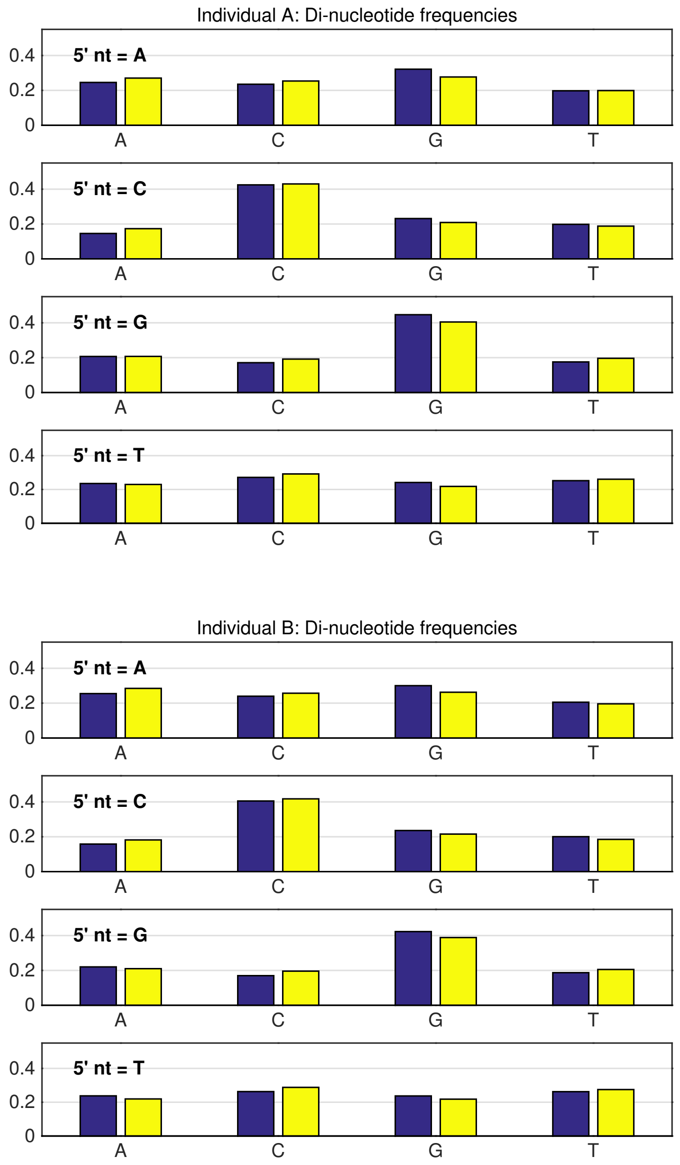

FIG. S10: Dinucleotide frequencies of VD andDJ insertions. Top panel - individual A, Bottom panel - individual B. A common dinucleotide Markov model accurately describes the sequence statistics of both VD and DJ insertions (reading VD sequences from the DNA top strand and the DJ insertions from the bottom strand).

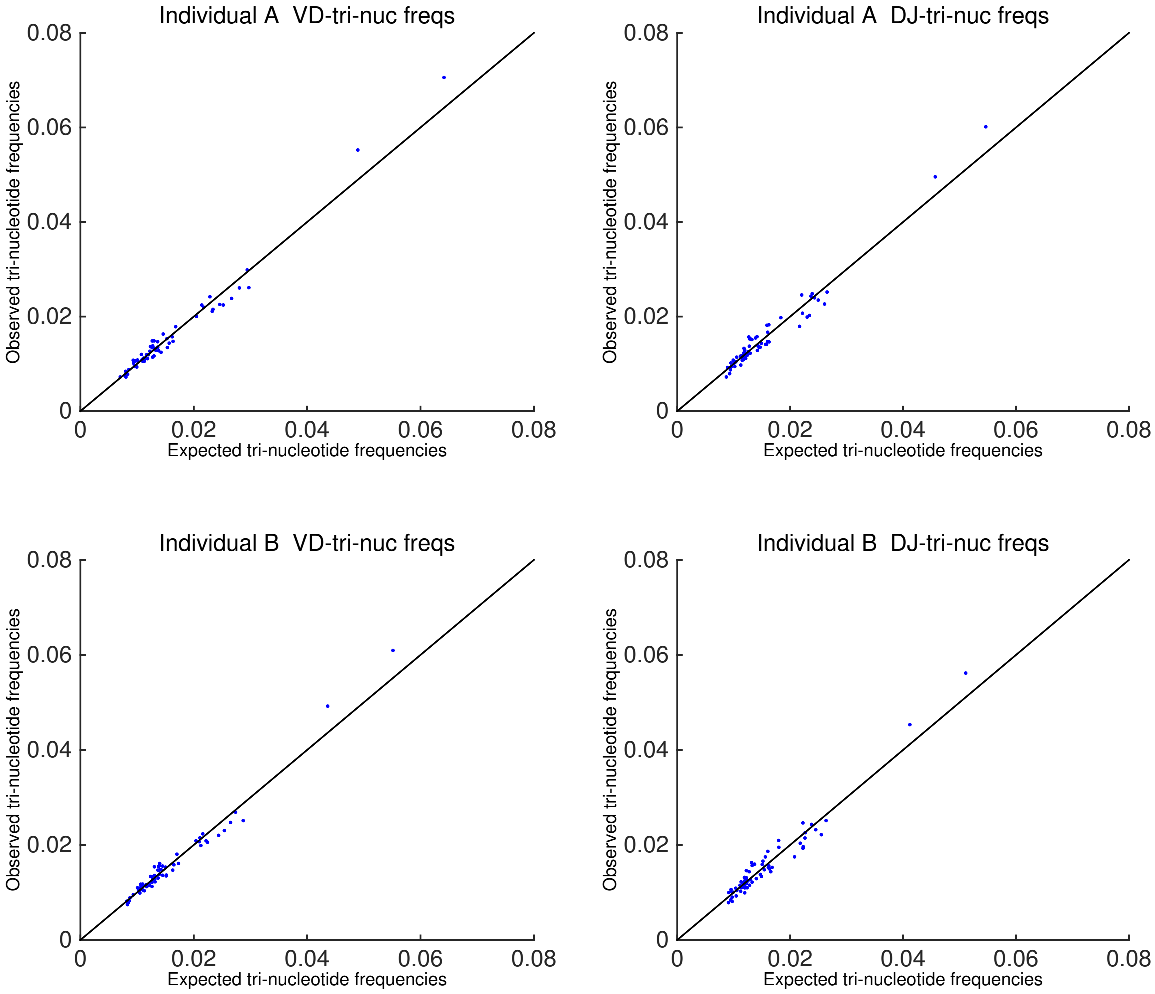

FIG. S11: Scatter plot of observed trinucleotide frequencies in VD and DJ insertions against their frequencies as predicted by the dinucleotide Markov model. The plot clearly indicates that three-base correlations are accurately reproduced by the dinucleotide Markov model for both individuals and both types of insertion.

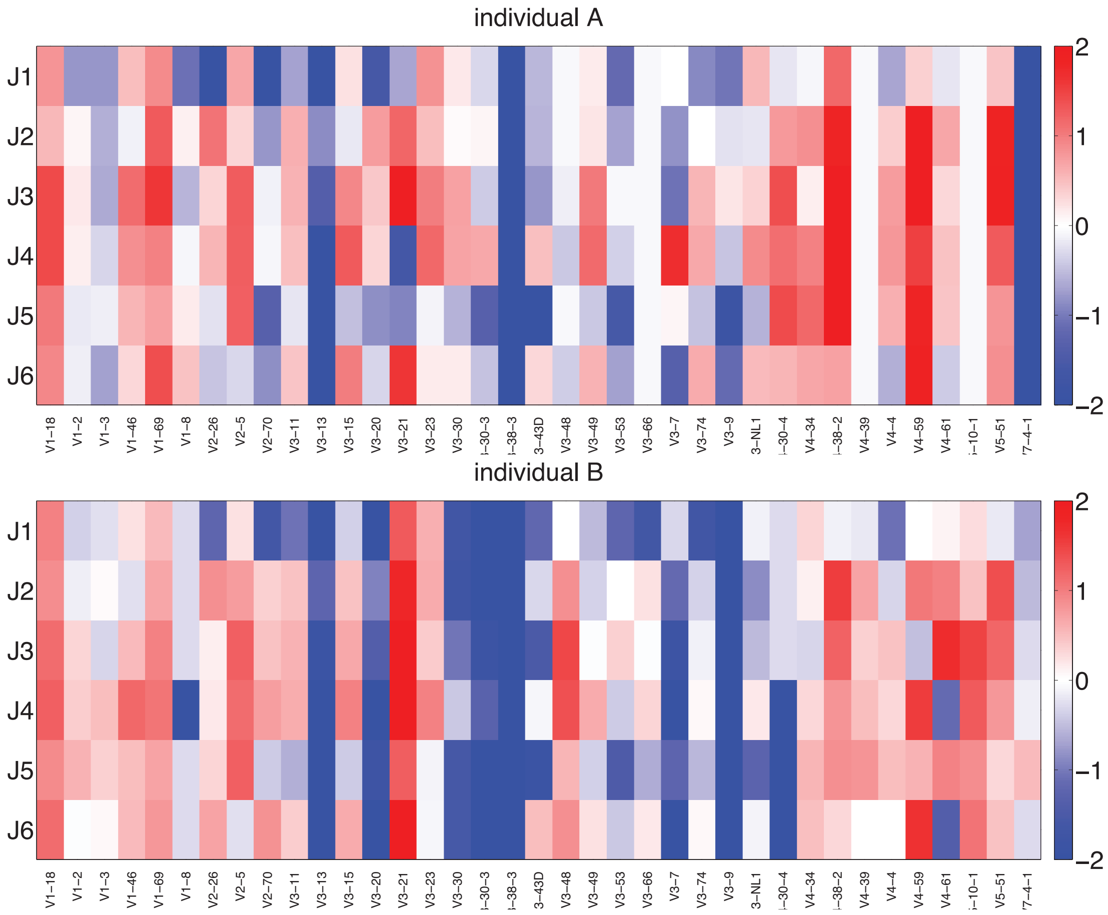

FIG. S12: Selection factors of pairs of V and J genes for both individuals (heat plot of the logarithm of the selection factors q_{VJ}). Again, only V genes with usage frequency greater than .01 for both individuals are shown. V selection pattern is different between individuals, but J pattern is more similar across genes.
